# Prediction of perioperative complications after robotic-assisted radical hysterectomy for cervical cancer using the modified surgical Apgar score

**DOI:** 10.1186/s12885-018-4809-4

**Published:** 2018-09-21

**Authors:** Seon Hee Park, Jung-Yun Lee, Eun Ji Nam, Sunghoon Kim, Sang Wun Kim, Young Tae Kim

**Affiliations:** Department of Obstetrics and Gynecology, Institute of Women’s Life Medical Science, Yonsei University College of Medicine, 50-1 Yonsei-ro, Seodaemun-gu, Seoul, 03722 South Korea

**Keywords:** Surgical Apgar score (SAS), Robotic-assisted radical hysterectomy, Cervical cancer

## Abstract

**Background:**

Although there has been marked development in surgical techniques, there is no easy and fast method of predicting complications in minimally invasive surgeries. We evaluated whether the modified surgical Apgar score (MSAS) could predict perioperative complications in patients undergoing robotic-assisted radical hysterectomy.

**Methods:**

All patients with cervical cancer undergoing robotic-assisted radical hysterectomy at our institution between January 2011 and May 2017 were included. Their clinical characteristics were retrieved from their medical records. The surgical Apgar score (SAS) was calculated from the estimated blood loss, lowest mean arterial pressure, and lowest heart rate during surgery. We modified the SAS considering the lesser blood loss typical of robotic surgeries. Perioperative complications were defined using a previous study and the Clavien-Dindo classification and subdivided into intraoperative and postoperative complications. We analyzed the association of perioperative complications with low MSAS.

**Results:**

A total of 138 patients were divided into 2 groups: with (*n* = 53) and without (*n* = 85) complications. According to the Clavien-Dindo classification, 49 perioperative complications were classified under Grade I (73.1%); 13, under Grade II (19.4%); and 5, under Grade III (7.5%); 0, under both Grade IV and Grade V. Perioperative complications were significantly associated with surgical time (*p* = 0.026). The MSAS had a correlation with perioperative complications (*p* = 0.047). The low MSAS (MSAS, ≤6; *n* = 52) group had significantly more complications [40 (76.9%), *p* = 0.01]. Intraoperative complications were more correlated with a low MSAS than were postoperative complications [1 (1.2%) vs. 21 (40.4%); *p* < 0.001, 13 (15.1%) vs. 25 (48.1%); *p* = 0.29, respectively]. We also analyzed the risk-stratified MSAS in 3 subgroups: low (MSAS, 7–10), moderate (MSAS 5–6), and high risks (MSAS, 0–4). The prevalence of intraoperative complications significantly increased as the MSAS decreased *p* = 0.01).

**Conclusions:**

This study was consistent the concept that the intuitive and simple MSAS might be more useful in predicting intraoperative complications than in predicting postoperative complications in minimally invasive surgeries, such as robotic-assisted radical hysterectomy for cervical cancer.

## Background

Telerobotic surgery has been introduced in the field of gynecology and contributed to great progress in surgical management. Since the introduction of radical hysterectomy using robots in patients with early-stage cervical cancer, robotic-assisted radical hysterectomy has been rapidly adopted in gynecologic oncology. Robotic-assisted radical hysterectomy yields a shorter surgical time, lesser blood loss, shorter hospital stay, faster recovery, less postoperative pain, and fewer complications than does traditional abdominal radical hysterectomy and laparoscopic-assisted radical hysterectomy [1–3]. Although there has been marked development in surgical techniques, there is no easy and fast method of predicting complications in minimally invasive surgeries. Predicting postoperative complications improves the quality of care by increasing patient satisfaction and reducing the readmission rate and medical resource wasting [4].

In 1953, the neonatal Apgar score was introduced and has brought a considerable change the prediction of neonatal outcomes [5]. Gawande et al. devised the surgical Apgar score (SAS) based on this concept and applied it to general or vascular surgery [6]. The SAS, a 10-point scoring system, comprises 3 components: estimated blood loss (EBL), mean arterial pressure (MAP), and heart rate (HR) during surgery. The SAS ranges from 0 to 10; a low SAS indicates poor outcomes as in the neonatal Apgar score.

Since its introduction, the SAS has been validated for its usefulness in surgical fields, including urologic, head and neck, neurosurgical, orthopedic, transplant, and gastrointestinal surgeries [7–15]. It has also been used in gynecologic oncology. Low SASs (≤4) were reported as a strong predictor of postoperative complications after cytoreduction for advanced ovarian cancer [16]. The SAS was applied to elderly patients who underwent non-laparoscopic surgery and was confirmed its usefulness [17]. Low SASs were associated with morbidity but were unable to predict postoperative complications in patients undergoing hysterectomy for malignancy [18]. This controversy exists in other studies. Most studies have reported that the SAS was useful in predicting postoperative complications; however, other studies could not confirm this association [9, 19, 20]. All previous studies on the SAS have been conducted in laparotomy surgery; no study has used this on minimally invasive surgeries, which are currently replacing traditional surgical approaches. We sought to evaluate whether the SAS could predict perioperative complications in patients undergoing robotic-assisted radical hysterectomy for cervical cancer.

## Methods

All patients with cervical cancer undergoing robotic radical surgery at our institution between January 2011 and May 2017 were included. All final diagnoses were confirmed by our gynecologic pathologists. Patients were excluded if they had incomplete medical records. Radical hysterectomy or trachelectomy (and/or bilateral pelvic lymph node dissection, paraaortic lymph node dissection, and/or bilateral salpingo-oophorectomy) was performed depending on the stage and fertility preservation of the patients. Data were retrieved from the patients’ medical records (inpatient and outpatient, pathologic, anesthetic, and surgical records). We collected data on the patients’ demographics, perioperative complications, and SAS.

Perioperative complications were defined as described by previous study and the Clavien-Dindo classification, and subdivided into intraoperative and postoperative complications [1]. Postoperative complications included fever (> 37.8 °C) for > 24 h postoperatively, urinary distention, ileus, vault bleeding, readmission within 30 days after surgery, lymphedema, nerve palsy, wound dehiscence, fistula, and peritonitis. Intraoperative complications included transfusion within 72 h after surgery, large blood loss amount (≥300 mL), and organ injury.

The SAS was calculated from the EBL, lowest MAP, and lowest HR. Because the EBL of the SAS was set for open surgery, applying it to minimally invasive surgeries was difficult. Thus, we applied the modified SAS (MSAS) based on the lesser blood loss typical of robotic surgeries (Table 1). The lowest HR and MAP were obtained from the anesthetic records. During surgery, the patients’ vital signs were recorded on an anesthesia record every 5 min. The EBL was obtained from the surgical records. The surgical team (surgeon, nurse, and anesthesiologist) evaluated blood loss after surgery and recorded it on the surgical note. We also analyzed the risk-stratified MSAS in 3 subgroups: low (MSAS, 7–10), moderate (MSAS, 5–6), and high risks (MSAS, 0–4).

**Table 1:** Original SAS and MSAS

	0 points	1 point	2 points	3 points	4 points
SAS					
EBL (mL)	> 1000	601–1000	101–600	≤100	_
Lowest MAP (mmHg)	< 40	40–54	55–69	≥70	_
Lowest HR (beats/min)	> 85	76–85	66–75	55–65	≤55
MSAS					
EBL (mL)	> 300	151–300	51–150	≤50	_
Lowest MAP (mmHg)	< 40	40–54	55–69	≥70	_
Lowest HR (beats/min)	> 85	76–85	66–75	55–65	≤55

*SAS* surgical Apgar score, *MSAS* modified surgical Apgar score, *EBL* estimated blood loss, *MAP* mean arterial pressure, *HR* heart rate

Clinical variables were compared using Student’s t-test as appropriate for univariate analysis. Categorical variables were compared using Chi-square test and Fisher’s exact test. Significantly associated variables were analyzed utilizing the linear-by-linear association test. Data were shown as means [± standard deviations (SDs)], medians (ranges), and numbers of patients (%), where applicable. For all statistical analyses, *p*-values < 0.05 were considered statistically significant. Statistical analysis was performed using SPSS version 23.0 (Chicago, Illinois, USA).

## Results

Between January 2011 and May 2017, 148 patients underwent robotic-assisted radical surgery for cervical cancer at our institution; 10 of them had incomplete medical records. Thus, a total of 138 patients were finally enrolled. Twenty-one patients (17.95%) underwent robotic-assisted radical trachelectomy, and 117 patients (82.05%) underwent RRH. In this cohort, 53 patients (38.4%) had a total of 67 perioperative complications; 27 patients (19.6%) had intraoperative complications; 38 patients (27.5%), postoperative complications; and 12 patients (8.7%), both. According to the Clavien-Dindo classification, 49 perioperative complications were classified under Grade I (73.1%); 13, under Grade II (19.4%); and 5, under Grade III (7.5%); 0, under both Grade IV and Grade V. Table 2 shows the perioperative complications details.

**Table 2:** The Clavien-Dindo classification and perioperative complications details

	Number	Percent
Clavien-Dindo classification^a^		
Grade I	49	73.1
Grade II	13	19.4
Grade III	5	7.5
Grade IV	0	0
Grade V	0	0
Perioperative complications^b^		
Intraoperative complications		
Bleeding (≥300 mL)	16	11.59
Transfusion	10	7.25
Bowel injury	1	0.72
Bladder or ureter injury	1	0.72
Postoperative complications		
Fever for > 24 h	17	12.32
Urinary distention	6	4.35
Ileus	3	2.17
Vault bleeding	2	1.45
Readmission at < 30 days	2	1.45
Lymphedema	2	1.45
Dysrhythmia	2	1.45
Nerve palsy	2	1.45
Wound dehiscence	1	0.72
Fistula	1	0.72
Peritonitis	1	0.72

^a^In relation to the total number of complications (*n* = 67)

^b^In relation to the total number of patients (*n* = 138)

The patients’ clinical characteristics and MSASs are summarized in Table 3. The mean age and body mass index (BMI) were not significantly different between the groups. Neoadjuvant chemotherapy (NAC) before surgery [4 (4.7%) vs. 3 (5.8%); *p* = 0.77] and surgical radicality were also not significantly different (*p* = 0.47). Perioperative complications were significantly associated with surgical time (*p* = 0.026). The SAS was also not significantly different; however, the MSAS was associated with perioperative complications (*p* = 0.047).

**Table 3:** Patients’ clinical characteristics and MSAS

	Complication		*P-*value
	No (*n* = 85)	Yes (*n* = 53)	
Age (years)	44.1 (±10.1)	43.1 (±9.8)	0.51
BMI (kg/m^2^)	22.9 (±3.0)	22.9 (±3.3)	0.98
Underlying disease (*n*)	17 (20.0)	7 (13.2)	0.31
Previous pelvic surgery (*n*)	35 (41.2)	18 (33.7)	0.40
Surgical time (min)	190.0 (±70.9)	221.0 (±90.0)	0.026
Preoperative Hb (g/dL)	13.0 (±1.20)	12.6 (±1.3)	0.10
Postoperative Hb (g/dL)	12.8 (±1.3)	11.0 (±1.3)	0.27
NAC (*n*)	4 (4.7)	3 (5.8)	0.77
Surgical procedure (*n*)			0.47
Radical trachelectomy (±BPLD and PALND)	12 (14.1)	9 (17.0)	
Radical hysterectomy (±BPLD, PALND, and BSO)	73 (85.9)	44 (83.0)	
SAS	7.4 (±1.1)	7.1 (±1.3)	0.27
MSAS	7.0 (±1.2)	6.5 (±1.8)	0.047

*MSAS* modified surgical Apgar score, *BMI* body mass index, *Hb* hemoglobulin, *NAC* neoadjuvant chemotherapy, *BPLD* bilateral pelvic lymph node dissection, *PALND* paraaortic lymph node dissection, *BSO* bilateral salpingo-oophorectomy, *SAS* surgical Apgar score

Data are presented as means (± standard deviations) or as numbers of patients (%)

Table 4 shows the correlation of the complications with a low MSAS (MSAS, ≤6). In the total cohort, 52 patients (36.7%) had an MSAS of ≤6, and 86 patients (62.3%) had an MSAS of > 6. We did not find an association between a low MSAS and age, BMI, underlying disease, previous pelvic surgery, NAC, and extensive surgical procedures. The preoperative hemoglobulin level was higher in the low MSAS group than in the high MSAS group [mean (SD): 13.2 (±1.2) vs. 12.6 (±1.2); *p* = 0.01]. The low MSAS group had significantly more complications [40 (76.9%), *p* = 0.01]. The intraoperative complications were more correlated with a low MSAS than the postoperative complications [1 (1.2%) vs. 21 (40.4%); *p* < 0.001, 13 (15.1%) vs. 25 (48.1%); *p* = 0.29, respectively].

**Table 4:** Characteristics of the patients with low MSASs

	MSAS of > 6 (*n* = 86)	MSAS of ≤6 (*n* = 52)	*P-*value
Age (years)	43.1 (±9.9)	44.7 (±10.1)	0.37
BMI (kg/m^2^)	22.7 (±2.9)	23.3 (±3.8)	0.27
Underlying disease (*n*)	15 (17.4)	9 (17.3)	0.98
Previous pelvic surgery (*n*)	36 (41.9)	17 (32.7)	0.29
NAC (*n*)	4 (4.7)	3 (5.8)	0.77
Radicality of surgery			0.33
Preoperative Hb (g/dL)	12.6 (±1.2)	13.2 (±1.2)	0.012
Perioperative complications (*n*)	13 (15.1)	40 (76.9)	0.01
Intraoperative complications (*n*)	1 (1.2)	26 (50)	< 0.001
Postoperative complications (*n*)	13 (15.1)	25 (48.1)	0.29

*MSAS* modified surgical Apgar score, *BMI* body mass index, *NAC* neoadjuvant chemotherapy, *Hb* hemoglobulin

Data are provided as means (± standard deviations) or as numbers of patients (%)

In the low MSAS group, perioperative complications occurred in 40 (76.9%) patients; intraoperative complications in 26 (50%) patients; and postoperative complications in 25 (48.1%) patients. Table 5 shows the association between a low MSAS and operative complications; a low MSAS was not significantly associated with perioperative [odds ratio (OR), 1.43; *p* = 0.36] and postoperative complications (OR, 0.711; *p* = 0.4). However, a low MSAS was a predictor for intraoperative complications (OR, 3.57; 95% confidence interval: 1.0–12.7; *p* = 0.039) (Table 5).

**Table 5:** Low MSASs (≤6) as a predictor for intraoperative complications

	Odds ratio	95% CI	*P-*value
For perioperative complications	1.43	0.7–3.1	NS
For intraoperative complications	3.570	1.0–12.7	0.039
For postoperative complications	0.711	0.3–1.6	NS

*NS* not significant, *CI* confidence interval

**Fig. 1:**
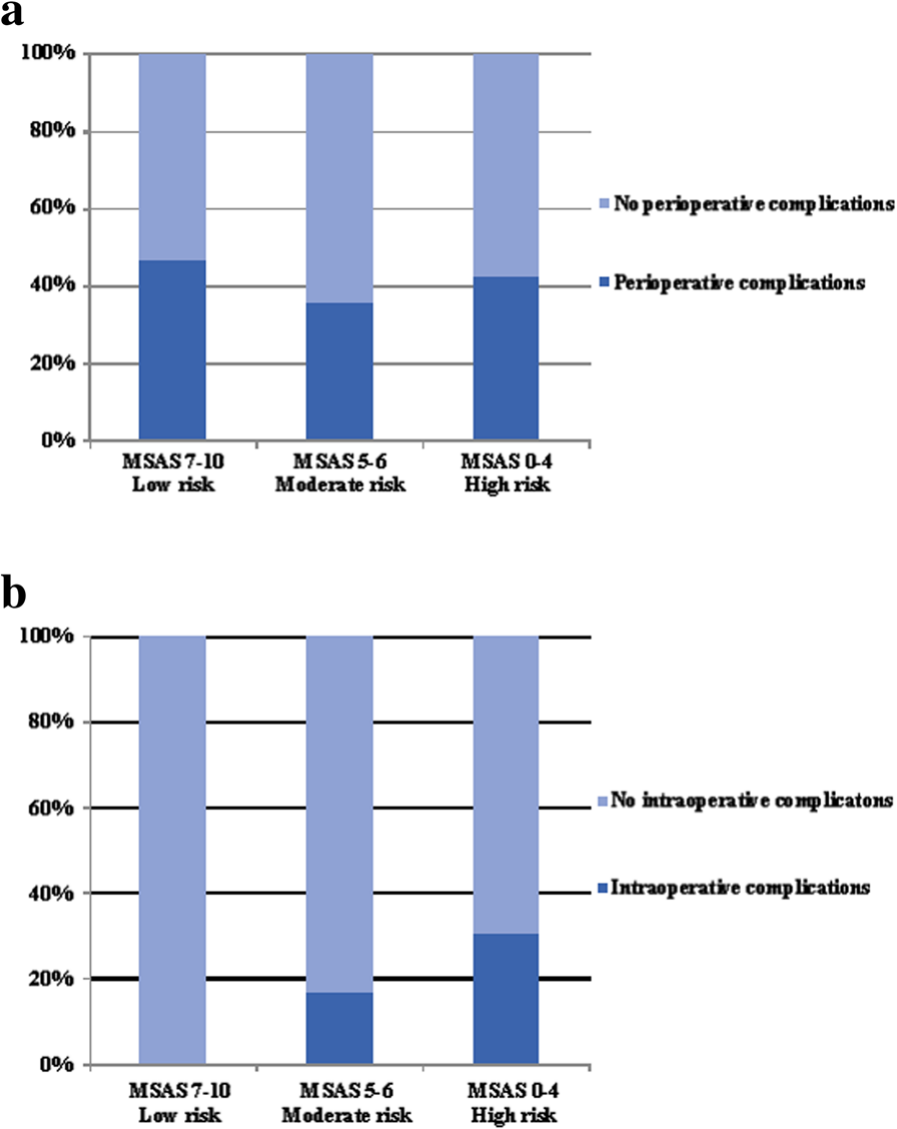
Linear-by-linear association test results of the perioperative and intraoperative complications with the risk-stratified MSAS. (**a**) Association between perioperative complications and risk-stratified MSAS; (**b**) association between intraoperative complications and risk-stratified MSAS. The patients with increasing risk-stratified MSASs have a higher incidence of intraoperative complications (**b**) (*p* = 0.01). However, this association was not found in the perioperative complications (**a**) (*p* = 0.98). MSAS, modified surgical Apgar score

The linear-by-linear association test results for the perioperative and intraoperative complications with the risk-stratified MSASs are described in Fig. 1. The number of patients with perioperative complications was 7/15 (46.7%) in the low-risk group, 32/90 (35.6%) in the moderate-risk group, and 14/33 (42.4%) in the high-risk group. Among the patients with intraoperative complications, 0/15 (0%) were included in the low-risk group; 15/90 (16.7%) in the moderate-risk group; and 10/33 (30.3%) in the high-risk group. We could not confirm the significance of the increased prevalence of perioperative complications with the decreased MSAS (*p* = 0.98). However, the intraoperative complications significantly increased as the MSAS decreased (*p* = 0.01). The patients with increasing risk-stratified MSASs had a higher incidence of intraoperative complications.

## Discussion

The current study showed that a low MSAS could predict perioperative complications in patients undergoing robotic-assisted radical hysterectomy. This supports the findings of previous studies that validated the usefulness of the SAS in predicting perioperative complications after major surgeries [16, 17]. The EBL scale in the SAS was slightly modified account for the lesser blood loss in robotic-assisted surgeries. A previous study also employed the modified EBL scale because of the larger blood loss and showed that the SAS could predict major postoperative complications and death [7]. The difference between the current and previous studies is that most of the latter defined low SASs as ≤4; conversely, we defined it as ≤6 [17]. We thought that the lesser blood loss, which is the strength of robotic-assisted radical hysterectomy, had influenced our results. Furthermore, our low MSAS definition reflects the risk assessment system of the Apgar score better, which is the basis of the SAS.

One study reported hospital readmissions within 30 days after discharge in 13.2% of 2455 patients in gynecologic oncology. Among them, 87% had gynecologic malignancies and 22.2% had cervical cancer [4]. These unplanned readmissions are more costly than planned hospitalizations and yield lower medical care quality. Therefore, clinicians strive to predict postoperative complications using preoperative and intraoperative assessment tools, such as the American Society of Anesthesiologists Physical Status Classification System (ASA classification) [21], Acute Physiology and Chronic Health Evaluation (APACHE) [22], and Physiological and Operative Severity Score for the enUmeration of Mortality and Morbidity (POSSUM) [23]. However, the ASA classification only reflects the patients’ preoperative physical status and therefore does not reflect the patients’ intraoperative conditions due to unexpected complications during surgery. Because of the complicated scoring system, the APACHE and POSSUM are not widely used in clinical practice for postoperative risk prediction [18, 24]. Complexity of surgical procedures, elevated serum glutamic oxaloacetic transaminase levels, higher ASA scores, and obesity were reported as risk factors for adverse events in gynecological cancer surgery [16, 25]. However, most patients with cervical cancer who can be treated surgically have no preoperative underlying diseases and do not have these preoperative risk factors. Therefore, intraoperative patient assessment is an important factor for predicting postoperative complications. For these reasons, surgeons need a useful tool to evaluate the patients’ condition during urgent surgeries so that patients can receive appropriate postoperative care.

The SAS could be easily applied in clinical practice because it is a simple and intuitive scoring system. This scoring system assesses intraoperative management using only 3 objective factors (i.e., EBL, lowest HR, and lowest MAP during surgery); the lower the score, the higher the postoperative complication risk. The SAS is easy to calculate, interpret, and apply. Because of these attractive strengths, many researchers have been working on the clinical application of this new risk assessment tool to predict postoperative complications. The original study was conducted on patients who underwent colectomy, and the subsequent validation studies were conducted on large samples in other surgical fields. The SAS was applied to 4119 general and vascular surgery patients and demonstrated its usefulness [26]. The past study confirmed the validity of the SAS in patients from a low-income country who underwent laparotomy [27]. Recently, a new surgical scoring system has been proposed that combines intraoperative assessment of the SAS and preoperative assessment of the ASA score to predict postoperative mortality [28, 29]. Immediate recognition of postoperative complications leads to careful postoperative management, prevents postoperative complications, and improves patient outcome [30].

Because the SAS reflects the intraoperative management status, low MSASs have a higher correlation with intraoperative complications. The patients with lower MSASs had a higher incidence of intraoperative complications. The previous study defined perioperative complications as blood transfusion after surgery and a large blood loss amount during surgery [16]. We classified perioperative complications into intraoperative and postoperative complications and defined intraoperative complications as blood transfusion, a large blood loss amount, and organ injury.

One of the interesting findings of our study is that only 1 patient received transfusion among the 16 patients with an EBL amount of ≥300 mL. Postoperative transfusion was associated with lower MSASs rather than was absolute blood loss amount. Three pillars of transfusion in surgical patients were reported: detection and treatment of preoperative anemia; reduction of perioperative blood loss; and harnessing and optimizing the patient-specific physiological reserve of anemia [31, 32]. In addition to these 3 factors, our results showed that proper intraoperative management is also important for transfusion of surgical patients.

Our study has some limitations. First, this was a retrospective study conducted at a single tertiary academic center, and data were collected only from patients with cervical cancer undergoing robotic surgery. Second, the HR and blood pressure were recorded every 5 min in anesthesia records. Not all values were recorded during surgery; however, according to the monitoring guideline for safe anesthesia, vital signs were measured at least every 5 min or more often, if necessary, so that blood pressure and HR can be accurately assessed [17, 33]. Finally, our results were derived from a single disease and single surgical procedure, with similar surgical radicalities.

Despite these limitations, to the best our knowledge, this is the first study to investigate the MSAS in patients undergoing robotic-assisted radical hysterectomy. Preoperative and intraoperative risk assessments are important for surgeons to provide adequate postoperative care for patients. The 3 parameters could be useful for intraoperative risk assessment in minimally invasive surgeries. For the MSAS to be generally accepted, further prospective studies should be performed in various institutions and on various diseases and other minimally invasive surgeries.

## Conclusions

This study was consistent the concept that the intuitive and simple MSAS might be more useful in predicting intraoperative complications than in predicting postoperative complications in minimally invasive surgeries, such as robotic-assisted radical hysterectomy for cervical cancer.

## Abbreviations

BMI: Body mass index
BPLD: Bilateral pelvic lymph node dissection
BSO: Bilateral salpingo-oophorectomy
EBL: Estimated blood loss
Hb: Hemoglobulin
HR: Heart rate
MAP: Mean arterial pressure
MSAS: Modified surgical Apgar score
NAC: Neoadjuvant chemotherapy
PALND: Paraaortic lymph node dissection
SAS: Surgical Apgar score

## References

[1] Weinberg L, Rao S, Escobar PF (2011). Robotic surgery in gynecology: an updated systematic review. Obstet Gynecol Int.

[2] Long E, Kew F. Patient satisfaction with robotic surgery. J Robot Surg. 2017. 10.1007/s11701-017-0772-3 29288373

[3] Zanagnolo V, Garbi A, Achilarre MT, Minig L (2017). Robot-assisted surgery in gynecologic cancers. J Minim Invasive Gynecol.

[4] Henretta MS, Scalici JM, Engelhard CL, Duska LR (2011). The revolving door: hospital readmissions of gynecologic oncology patients. Gynecol Oncol.

[5] Apgar V. A Proposal for a New Method of Evaluation of the Newborn Infant. Originally published in July 1953, volume 32, pages 250-259. Anesth Analg. 2015, 120(5):1056–9. 10.1213/ANE.0b013e31829bdc5c 25899272

[6] Gawande AA, Kwaan MR, Regenbogen SE, Lipsitz SA, Zinner MJ (2007). An Apgar score for surgery. J Am Coll Surg.

[7] Prasad SM, Ferreria M, Berry AM, Lipsitz SR, Richie JP, Gawande AA, Hu JC (2009). Surgical apgar outcome score: perioperative risk assessment for radical cystectomy. J Urol.

[8] Orberger M, Palisaar J, Roghmann F, Mittelstadt L, Bischoff P, Noldus J, Loppenberg B (2017). Association between the surgical Apgar score and perioperative complications after radical prostatectomy. Urol Int.

[9] Ettinger KS, Moore EJ, Lohse CM, Reiland MD, Yetzer JG, Arce K (2016). Application of the surgical Apgar score to microvascular head and neck reconstruction. J Oral Maxillofac Surg.

[10] Hsu SY, Ou CY, Ho YN, Huang YH (2017). Application of surgical Apgar score in intracranial meningioma surgery. PLoS One.

[11] Sakan S, Pavlovic DB, Milosevic M, Virag I, Martinovic P, Dobric I, Davila S, Peric M (2015). Implementing the surgical Apgar score in patients with trauma hip fracture. Injury.

[12] Wied C, Foss NB, Kristensen MT, Holm G, Kallemose T, Troelsen A (2016). Surgical apgar score predicts early complication in transfemoral amputees: retrospective study of 170 major amputations. World J Orthop.

[13] Stoll WD, Taber DJ, Palesch SJ, Hebbar L (2016). Utility of the surgical Apgar score in kidney transplantation: is it feasible to predict ICU admission, hospital readmission, length of stay, and cost in this patient population?. Prog Transplant.

[14] Eto K, Yoshida N, Iwatsuki M, Kurashige J, Ida S, Ishimoto T, Baba Y, Sakamoto Y, Miyamoto Y, Watanabe M (2016). Surgical Apgar score predicted postoperative morbidity after Esophagectomy for esophageal Cancer. World J Surg.

[15] Giugliano DN, Morgan A, Palazzo F, Leiby BE, Evans NR, Rosato EL, Berger AC (2017). Surgical Apgar score (SAS) predicts perioperative morbidity, mortality, and length of stay in patients undergoing esophagectomy at a high-volume center. J Surg Oncol.

[16] Zighelboim I, Kizer N, Taylor NP, Case AS, Gao F, Thaker PH, Rader JS, Massad LS, Mutch DG, Powell MA (2010). “surgical Apgar score” predicts postoperative complications after cytoreduction for advanced ovarian cancer. Gynecol Oncol.

[17] Kurata K, Chino Y, Shinagawa A, Kurokawa T, Yoshida Y (2017). Surgical Apgar score predicts 30-day morbidity in elderly patients who undergo non-laparoscopic gynecologic surgery: a retrospective analysis. Int J Surg.

[18] Clark RM, Lee MS, Alejandro Rauh-Hain J, Hall T, Boruta DM, del Carmen MG, Goodman A, Schorge JO, Growdon WB (2015). Surgical Apgar score and prediction of morbidity in women undergoing hysterectomy for malignancy. Gynecol Oncol.

[19] Terekhov MA, Ehrenfeld JM, Wanderer JP (2015). Preoperative surgical risk predictions are not meaningfully improved by including the surgical Apgar score: an analysis of the risk quantification index and present-on-admission risk models. Anesthesiology.

[20] Hyder JA (2016). Predilection for poor prediction with the surgical Apgar score. Anesthesiology.

[21] Wolters U, Wolf T, Stutzer H, Schroder T (1996). ASA classification and perioperative variables as predictors of postoperative outcome. Br J Anaesth.

[22] Knaus WA, Zimmerman JE, Wagner DP, Draper EA, Lawrence DE (1981). APACHE-acute physiology and chronic health evaluation: a physiologically based classification system. Crit Care Med.

[23] Copeland GP, Jones D, Walters M (1991). POSSUM: a scoring system for surgical audit. Br J Surg.

[24] Vincent C, Moorthy K, Sarker SK, Chang A, Darzi AW (2004). Systems approaches to surgical quality and safety: from concept to measurement. Ann Surg.

[25] Kondalsamy-Chennakesavan S, Bouman C, De Jong S, Sanday K, Nicklin J, Land R, Obermair A (2009). Clinical audit in gynecological cancer surgery: development of a risk scoring system to predict adverse events. Gynecol Oncol.

[26] Cihoric M, Toft Tengberg L, Bay-Nielsen M, Bang Foss N (2016). Prediction of outcome after emergency high-risk intra-abdominal surgery using the surgical Apgar score. Anesth Analg.

[27] Ngarambe C, Smart BJ, Nagarajan N, Rickard J (2017). Validation of the surgical Apgar score after laparotomy at a tertiary referral Hospital in Rwanda. World J Surg.

[28] Kinoshita M, Morioka N, Yabuuchi M, Ozaki M (2017). New surgical scoring system to predict postoperative mortality. J Anesth.

[29] Jering MZ, Marolen KN, Shotwell MS, Denton JN, Sandberg WS, Ehrenfeld JM (2015). Combining the ASA physical classification system and continuous intraoperative surgical Apgar score measurement in predicting postoperative risk. J Med Syst.

[30] Ghaferi AA, Birkmeyer JD, Dimick JB (2009). Complications, failure to rescue, and mortality with major inpatient surgery in medicare patients. Ann Surg.

[31] Richards T, Musallam KM, Nassif J, Ghazeeri G, Seoud M, Gurusamy KS, Jamali FR (2015). Impact of preoperative Anaemia and blood transfusion on postoperative outcomes in Gynaecological surgery. PLoS One.

[32] Spahn DR, Shander A, Hofmann A (2013). The chiasm: transfusion practice versus patient blood management. Best Pract Res Clin Anaesthesiol.

[33] Kurosawa S (2012). Anesthesia in patients with cancer disorders. Curr Opin Anaesthesiol.

